# Saudi high school students’ attitudes and barriers toward the use of computer technologies in learning English

**DOI:** 10.1186/2193-1801-3-460

**Published:** 2014-08-23

**Authors:** Ahmed Abdulateef Sabti, Rasha Sami Chaichan

**Affiliations:** Universiti Kebangsaan Malaysia (UKM), Selangor, Malaysia

**Keywords:** Students’ attitudes, Using e-learning, Gender differences

## Abstract

This study examines the attitudes of Saudi Arabian high school students toward the use of computer technologies in learning English. The study also discusses the possible barriers that affect and limit the actual usage of computers. Quantitative approach is applied in this research, which involved 30 Saudi Arabia students of a high school in Kuala Lumpur, Malaysia. The respondents comprised 15 males and 15 females with ages between 16 years and 18 years. Two instruments, namely, Scale of Attitude toward Computer Technologies (SACT) and Barriers affecting Students’ Attitudes and Use (BSAU) were used to collect data. The Technology Acceptance Model (TAM) of Davis (1989) was utilized. The analysis of the study revealed gender differences in attitudes toward the use of computer technologies in learning English. Female students showed high and positive attitudes towards the use of computer technologies in learning English than males. Both male and female participants demonstrated high and positive perception of Usefulness and perceived Ease of Use of computer technologies in learning English. Three barriers that affected and limited the use of computer technologies in learning English were identified by the participants. These barriers are skill, equipment, and motivation. Among these barriers, skill had the highest effect, whereas motivation showed the least effect.

## Introduction

The significance of English as medium of instruction for many communities around the world indicates that the demand to learn English has considerably increased. Crystal (2003: 3) observed that, “English has become the language of international business, diplomacy, trade, computer and even science, and is taught as a foreign language in more than 100 countries around the world”. Despite the diversity of the languages in Asia, such as Arabic, Malay, Urdu, Tamil, and Mandarin, English remains the lingua franca either for communication with each other in the same community or interaction with others from other communities (McArthur 2003). The demand to learn English is high among people whose mother tongue is not English. Hence, a high level of competence in the English language is sought by non-native English speakers because of its crucial role in the development and improvement of their skills (Mercer and Swann 1996).

Nowadays, the emergence of new technological advances, such as computers and the Internet, has led to the gradual development of new teaching and learning methods in English as a Second Language (ESL) and English as a Foreign Language (EFL) classrooms. These technologies have opened a whole new dimension in the investigation of the issue of teaching effectiveness. Kabilan (2009) indicates that the rapid revolutionary advances of computer technologies have led to extreme changes in education generally and language learning in particular, including reforming the curricular and new ways of literacy and pedagogy designs in English language learning.

The Ministry of Education of Saudi Arabia launched the Computer and Information Center (CIC) in 1996 to provide ICT services for ICT information and employment in education. The Ministry of education shows a considerable interest in this aspect to create educational plans and programs. However, despite the good intention of the education plans and programs to enhance the educational process, the government e-learning policies have remained ambiguous and lacking in terms of the goal of providing training, assistance, or appropriate guidance to attain the targets or fulfill the aims. These issues are similar to those encountered during the implementation of the UK ICT policy, a policy that referred to computers as “learning tools”, but did not define how these tools should be used to deliver this purpose (Blamire and Balanskat 2002). These observations highlight the idea that teachers should be trained, assisted, and guided on how to use and deal with these technologies to encourage their students to perform their tasks and raise their awareness regarding the use of these technologies for the benefit of the educational process.

Although schools in Saudi Arabia seek progress in relation to the development of educational process by providing the technical devices, one of the most significant aspects of the problem that students encounter in learning English is reliance on the traditional model. According to Rhema and Miliszewska (2010), this model of learning mainly depends “on face-to-face interactions in, and outside of classroom between students and teachers, and learning activities” (2010: 427). Moreover, students still rely on the textbooks prescribed by English teachers, which are distributed in the form of handouts. This may provide less motivation, self-confidence, and interest to practice and perform English learning activities in the classroom.

Another significant aspect of the problem is the insufficient exposure to computer technologies, which deters full utilization of computer technology devices by many Saudi EFL learners. Most students have minimal or no experience in using computers and those who know how to use computers employ these merely for entertainment and communication. Information communication technology (ICT) implementation without preparation and training on how to deal and use these technology tools properly might affect the learning process. The successful use of technologies can be largely detected in the attitudes and willingness of students to use these technology tools, which could lead to the successful utilization of these tools in the classroom. Albirini (2006) supports this view and states that the attitudes and willingness of learners toward using technology mainly value the successful use of technology in the educational settings.

## Literature review

### Technology Acceptance Model (TAM)

The current research paper is based on the Technology Acceptance Model (TAM) proposed by Davis (1989). He states that the influence of the external variables on technology acceptance is mediated by two individual beliefs, namely, (1) perceived usefulness (PU) and (2) perceived case of use (PEOU). Davis et al. (1989) define PU as “the prospective user's subjective probability that using a specific application system will increase his/her job performance within an organizational context, whereas PEOU is the degree to which the prospective user expects the target system to be free of effort”. To trace its first adoption, in line with (Schepers and Wetzels 2007; Mathieson 1991; Amoako-Gyampah and Salam 2004) that Davis (1989) is the initial one in developing TAM as an endeavor to adapt Theory of Reasoned Action (TRA) into an appropriate model to test and investigate users' acceptance of information technology. The aim of TAM is to clarify and explain user acceptance of computers in general, and identifies user behavior across a wide range of end-user computing technologies and user populations in particular (Davis et al. 1989).

**Figure 1:**
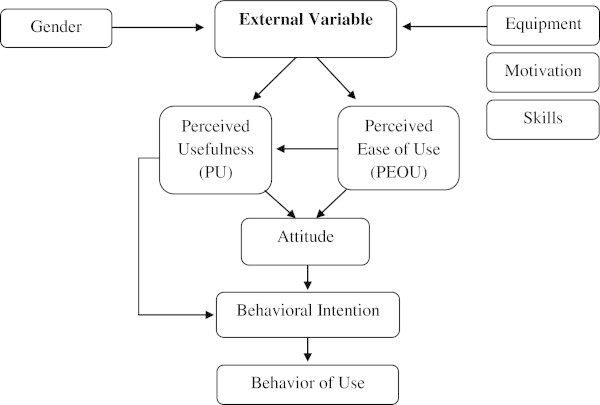
**Theoretical framework of Technology Acceptance Model (TAM) (Davis** 1989 **).**

Figure 1 illustrates the relationships in TAM that affect user acceptance of information technology. TAM is the most cited model in the study of user acceptance and use of technology.

A study carried out by Bulut and AbuSeileek (2007), which involved 112 Saudi university students who study English at King University. The study investigated the attitudes toward the integration of Computer Assisted Language Learning (CALL) into the university syllabus, which aimed at enhancing the learning of the four skills in English. Three instruments were used to collect data, namely, instructional software, material such as CD-ROMs, electronic dictionaries, search engines, online materials, online chat, and achievement test at the end of the course. The findings revealed that the respondents showed positive attitudes towards the general use of CALL in learning English. Besides, the participants also exposed positive attitudes towards the specific use of CALL in learning the four skills of English language. Furthermore, the study exhibited the resistance of the students to the application of CALL in learning.

Another study by Al Shammari (2007) researched the Saudi EFL students’ reactions towards CALL at Institute of Public Administration (IPA) in Saudi Arabia. The study based on a survey of questionnaires as tools for data collection that distributed to 1465 participants who were undergraduate learners of English in four campuses in Saudi Arabia. Among the respondents, 1310 were males and 155 were females. The results identified the general attitudes of the participants toward the use of CALL in learning English as well as certain gender-based discrepancies among the participants in each university campus. Furthermore, the findings showed positive attitudes toward the use of CALL as an active tool to learn English, with females showing more positive attitudes than males toward the use of CALL to learn various English language skills.

Alaugab (2007) examined the attitudes of female faculty members and students toward the adoption and acceptance of online instruction, the benefits of online instruction implementation, and to explore the significant barriers that could prevent the effective implementation of online instruction. The sample of the study comprised 310 female faculty members and students from two female institutions in Saudi Arabia. The findings indicated that female faculty members and students showed positive attitudes toward online instruction, as well as positive perceived usefulness and the most significant benefits of online instruction. In contrast, several barriers were revealed that could prevent the implementation of online instruction. The study concluded that female faculty members have the tendency to teach online courses and female students preferred to be involved in courses under an online environment.

Numerous studies have investigated the use of online materials and techniques in the teaching and learning of languages including English. Kung (2005) focused on the use of online websites as a proficient strategy to improve the English reading skills of foreign learners. The study examined the reactions and perceptions of 48 students in English reading class at the College of Language in Southern Taiwan. The students were asked to use websites in learning while doing tasks. The study adopted the mixed method and used several instruments, such as questionnaires, interviews, documents, and assignments to collect data. The results indicated that the majority of the participants (64%) reacted positively and had positive attitudes and perceptions with regard to the use of electronic sources to help develop their English reading skills, knowledge, and vocabulary. Among the participants, 89% stated that these online websites aided them in accomplishing their reading tasks properly. Only a few of the participants (9%) had no positive reactions toward the use of online websites.

A study by Lim and Zhong Shen (2006) explored the effectiveness of CALL in EFL learning among Korean TAFE College students. The aim of study is identify the effect of CALL on teaching and learning English reading skills in the classroom via comparison between learning reading with CALL and conventional learning reading classes in which technology is almost absent. The study population consisted of 74 first year English students grouped into two. The first group learned to read with CALL, whereas the second group learned without CALL for one semester. The results demonstrated that students who adopted CALL in their reading classes were more confident and had positive ideas compared with their counterparts who were in the conventional learning class. Students in the first group had more positive attitudes and higher perceived usefulness of information technology than students in the traditional classes because the learning environment of classes with CALL was enhanced with more materials, such as websites, activities, and tasks, that were not available in group two classes.

Xiong (2008) conducted a mixed-method study (quantitative and qualitative) on the use of CALL in learning English. The results of the study revealed valuable information on the barriers and obstacles to the use of CALL that affected the participants. The barriers include technical support, heavy workload, difficulties in the adaptation of using such technologies, the frequent breakdown of computers, their limited professional skills in using computers, and other barriers related to Internet access and connection. The study disclosed that such these barriers could affect and restrict the students’ use of CALL in learning English.

Almuqayteeb (2009) demonstrated some variables and obstacles that influence the learners’ use of technology, such as computers and the Internet. The female participants claimed that they encountered some problems in using computers to teach English in class. The variables were of technical, physical, and administrative nature, such as the unavailability of sufficient technical support, proficient training on how to use the computers purposefully, absence of facilities or equipment, and the lack of encouragement from their administration. Other factors reported by the participants were environmental, such as the lack of moral, physical, technical, and professional supports. Finally, the researcher also pointed out that the technical and professional skills that might affect and limit the students’ use of technology.

Saltourides (2009) stated that the study implemented by Stepp-Greany (2002) to investigate how Spanish college students of English could grasp and learn English while employing technological tools like computers in order to identify the role of computers in improving their learning abilities. The findings, which were deduced from a comprehensive scanning of certain Internet activities and CDs, explained the process through which such technological tools helped English learners develop their passive skills (listening and reading). Nevertheless, the results revealed that the English learners did not benefit from these technologies in developing their productive skills (speaking and writing). The researcher assumed that, “the students' ability, their previous experience with technology, previous background in Spanish and the character types of the students were not taken account of, so such results could not be generalized to other classes where technology is used, since they were not statistical significant results” (Saltourides 2009: 57).

In conclusion, most of these previous studies dealt with university students. This current study however, aims to deal with the level of high school students to investigate the attitudes of Saudi Arabia learners and also to examine the barriers that affect and limit the use of computer technologies in learning English.

## Objective

This study is conducted to explore EFL Saudi students’ attitudes towards e-learning integration and the perceived barriers encountered by students in learning English at the intermediate public schools during the implementation of e-learning in educational institutions in the Kingdom of Saudi Arabia. The Saudi Arabia government established the National Center of E-learning and Distance Learning (ELC) with a cost of more than 47 million, it is necessary to examine and investigate students’ and teachers’ attitudes, readiness and acceptance. With Government’s financial support for e-learning and the lack of studies on e-learning use have highlighted the necessity of examining the attitudes and readiness of both students and teachers of the English language. Therefore, this has emphasized the urgency of the need to detect the weaknesses and strengths of this implementation to avoid the failure of this project and ensure that the large amount of money can be used properly to develop the criteria and implement these criteria in schools. These constitute the context by which the current study attempt to tackle the major issues facing EFL learners in the implementation of e-learning in public schools and to detect the weaknesses and strengths of this implementation to avoid failure and financial losses.

## Research questions

1. What are the attitudes of Saudi students toward the use of computer technologies in learning English?
2. What are the possible barriers that affect the attitudes of students and limit their actual usage of computer technologies in learning English?

## Methodology

**Figure 2:**
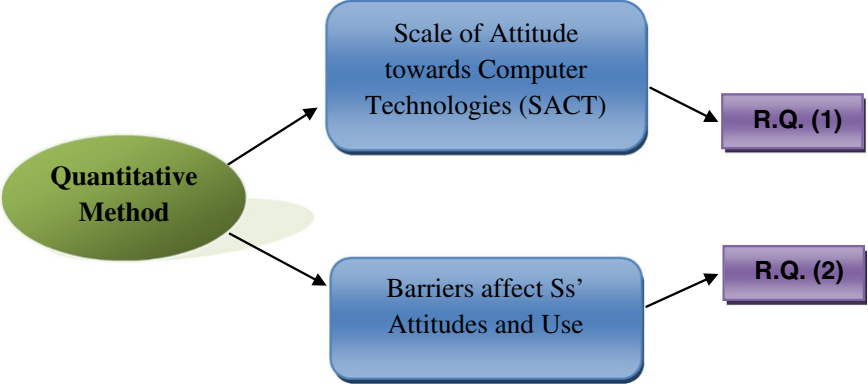
**Research design of quantitative method for the two instruments.**

The current research was based on the quantitative approach to investigate the attitudes of Saudi students toward the integration of e-learning in Saudi Arabia schools, in addition to the barriers that affect and limit their use of computer technologies in learning English. Figure 2 provides a clear picture of the current research design.

In this paper, the gender, race, and age of participants were controlled as demographic variables. The sample of study comprised 30 Saudi EFL students from a Saudi Arabia public school in Kuala Lumpur. Among the participants, 15 were males and 15 were females whose ages ranged from 16 years to 18 years. Permission to conduct the survey was obtained and granted by the director of the selected school.

Two instruments were used to collect the data in this study. The instruments were adopted from the study conducted by Elzaalouk (2010) in the National University of Malaysia (UKM). These two instruments were designed to collect a large quantity of information related to the subjects’ informational background, attitudes, and barriers that influence the implementation of such technologies in learning English.

The first instrument, *‘Attitudes toward Using Computer Technologies in Learning English’* consisted of 24 items. These items were built to discover the attitudes of students toward using computer tools in learning English. Owing to its value in collecting the data, it is needed to answer the first research question in the current study. Moreover, the instrument is utilized to obtain the data from the subjects regarding their attitudes toward the various patterns of computer and Internet applications in learning English.

The second instrument, ‘*Barriers that Affect Subjects' Attitudes and Limit their Use of Computer Technologies’* consisted of 10 items. This instrument refers to most influential factors obtained through the responses of participants to these items. These items include the barriers that could possibly affect the attitudes of the subjects and limit their actual use of computer technologies to learn English.

## Data analysis

The data were analyzed quantitatively. Two aspects of this study were analyzed using the Numerical Package for the Social Science Software (SPSS) version 20. First, the attitudes of students toward the use of technological tools in learning English were analyzed using Independent-Sample T-Test to determine the gender-based attitudes of Saudi Arabia students. Independent-Sample T-Test effectively determines the similarity and difference of attitudes between male and female participants toward the use of technological tools in learning English. Second, One-Way ANOVA was used to analyze the barriers that affect the attitudes of students toward the use of technology.

## Results

The results of this research paper are classified into two sections. The first section pertains to gender attitudes toward the application of computer technologies in learning English. The second section discusses the barriers that affect the students’ usage of technology in learning English.

**Figure 3:**
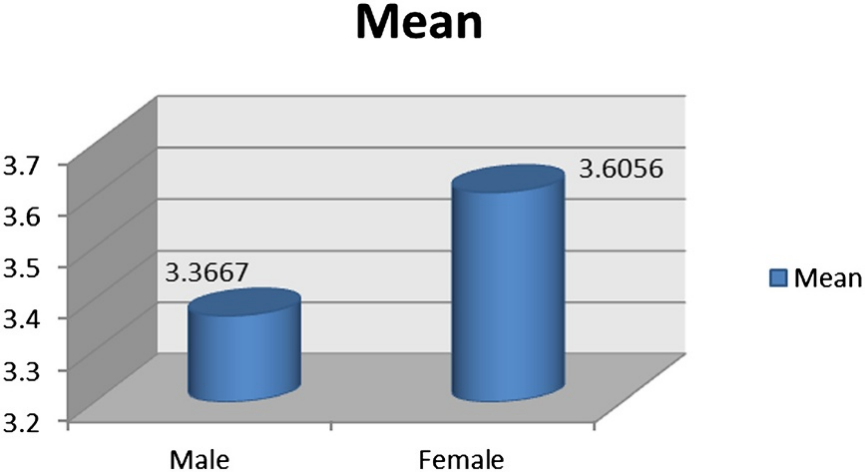
**Attitudes between male and female participants toward the use of technological devices in learning English.**

Figure 3 shows the results of the Independent Sample T-Test on gender differences in attitudes of Saudi Arabia students toward the use of technological devices in learning English. The analysis of this section also presents the subscale results for PU and PEOU.

**Figure 4:**
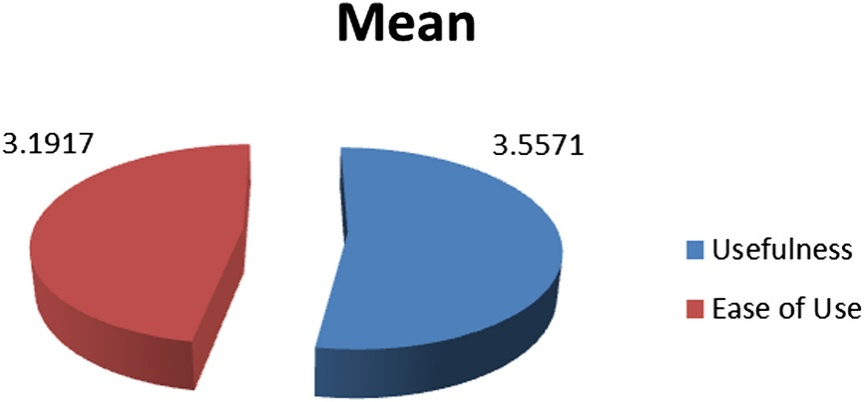
**Perception of Usefulness ‘PU’ and Perceived Ease of Use ‘PEOU’ toward the use of computer technologies in learning English.**

The results revealed that both of male and female participants showed higher tendency to use more technological tools in their schools. Although male and female participants had high attitudes, female participants demonstrated higher attitudes (3.6056) than their male counterparts (3.3667). These results imply that female participants had more positive attitudes toward the use of technology to learn English in their schools than males. The results also disclosed that participants showed highly positive Perception of Usefulness ‘PU’ (M = 3.5571) and Perceived Ease of Use ‘PEOU’ (M = 3.1917) on computer technologies to learn English. This highlights the interest of the participants to use computers tools in learning English (Figure 4).

**Figure 5:**
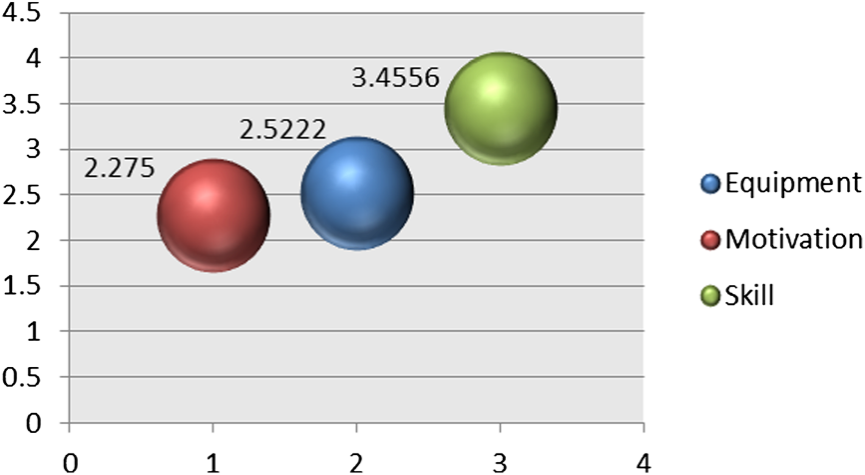
**Detected barriers (equipment, motivation and skill) that affect and limit the students’ use of computer tools in learning English.**

The second section presents the One-Way ANOVA analysis of the barriers that affect and limit the students’ use of computer technologies in learning English. Three barriers were detected that affected Saudi Arabia students, namely, equipment, motivation and skill. The values of first two barriers, equipment and motivation, were almost equal, indicating that the effect on the students is approximately at the same level (equipment 2.5222, motivation 2.2750). Besides that, these results also revealed the shortage of knowledge and experience on how to use the computer tools due to the higher barrier was skill (3.4556). This barrier (Skill) affected students more than the other two barriers (Figure 5). Skill refers to the proficiency, knowledge, and ability of students on dealing with these tools. Therefore, the respondents lack the experience and adequate information regarding computer devices in learning English. Figure 5 demonstrates the results of the second section.

## Discussion

### Attitudes toward using computer technologies in learning english

Studies on Saudi EFL learners reported that the responses of learners were mostly positive toward the utilization of computer and the Internet in learning English. The learners expressed that technology-based classrooms improved their learning. The overall mean scores of the attitudes toward computer technologies were 3.3667 for male participants and 3.6056 for female participants out of 5. This probably indicates that participants had showed high level of recognition of the significance of employing computer technologies in the process of learning English. The results of the current study are in accord with the findings of the study conducted by Kung (2005). The study of Kung demonstrated the majority of the subjects (64%) had the positive attitudes, reactions and perceptions of the fruitfulness of online websites as helpful materials in developing their reading skills, knowledge, and vocabulary. The findings of the current study also concur with the study of Bulut and AbuSeileek (2007), who demonstrated the positive ideas and reactions toward the general use of CALL in learning English of 112 Saudi university students of English at King Saud University. Similarly, the findings of Al Shammari (2007) support the results of the present study. He proved that Saudi EFL students positively support the use of CALL software as an effective technique to develop the process of learning English.

Results from the Independent Sample T-Test showed a considerable difference in the general perceptions of the participants toward the use of computer technologies. Gender took a significant place in this study that considerably influenced the overall attitudes of the participants. Female subjects showed more positive and stronger overall attitudes toward the use of computer technology in the process of learning English than the male subjects. These findings are similar to those of Al Shammari (2007), who pointed out that gender had an important role in influencing the attitudes of the subjects. Specifically, the present study revealed that female participants proved more positive attitudes than male participants toward CALL software use in learning English.

Furthermore, the findings also disclosed the participants’ attitudes of computer technology in terms of perceived usefulness, and perceived Ease of Use subscales. The participants’ scores of perceived usefulness were 3.5571 and scores of perceived Ease of Use were 3.1917. The findings exposed that the participants had high positive computer technology perceived usefulness and perceived Ease of Use subscales towards using computer technologies in learning English. The high levels of computer technology PU and PEOU indicated that the students achieved a high degree of perception on the usefulness of computer technology and a high level of interest to use such computer technologies. These results are consistent with the findings of Lim and Zhong Shen (2006) and Alaugab (2007). They revealed that participants presented positive computer technology attitude subscales, particularly their high levels of PU and recognition of the benefit of computer technologies and online instruction.

### Barriers affecting the attitudes of students and limiting their use of computer technologies in learning english

With reference to the second question attempted in this research i.e. the obstacles that learners encountered. The findings showed some barriers that influence the attitudes of participants and impede their use of online resources and the Internet in learning the English language. The barriers were classified into three groups, namely, the lack of equipment, lack of skills, and lack of motivation. The most influential obstacles that were identified by the subjects as shaping their reactions and restricting their use of computer technologies were external barriers, such as the lack of equipment and skills. These two types of barriers are related to the extent that affected learner’s stands and restricted their use of computer technologies. These two barriers were attributed to the external or physical environment. Nevertheless, the barriers that affected the attitudes of the participants and restricted their use of technologies were essential barriers, including the lack of motivation. However, the last group ‘motivation’ had low effect on the attitudes of the participants and the use of computer technologies. This clearly refers to the positive inner aspects that participants have, in addition to the encouragement given to the participants for the employment of technologies in learning English.

Among the three groups of barriers, the participants identified the lack of skill (score of 3.4556 of 5) as the most influential barrier on participants’ attitudes and restricting their use of computer technologies. The findings are inconsistent and generally contradictory with those of Kung (2005), who declared that the lack of the equipment is the most identified obstacle. The same findings are in accordance with those presented by Kung (2005), Alaugab (2007) and Almuqayteeb (2009), since they all discovered that the lack of equipment was reported as a barrier influencing the subjects' reactions and restricting the use of computer technologies. In the case of the current study, however, the lack of skill has been identified as the most important obstacle influencing the Saudi EFL learners' stands and restricting the use of computer technologies in learning English.

The group of the external obstacles identified and confronted by the Saudi EFL learners who participated in the current study was the lack of skills in employing the computer technologies. The skill barrier score was 3.4556, indicating that the participants’ lack of professional and technical skills slightly influenced their stands and restricted their use of computer technologies. Some researchers (Xiong 2008 and Almuqayteeb 2009) agreed that the lack of technical and professional skills in using computer technologies including the Internet considerably influence the attitudes of learners and restrict the application of computer technologies in learning English.

With regard to the final type of obstacles identified by the participants in the present study and classified under the internal type of obstacles, the study findings demonstrated that this obstacle (motivation) was the least significant one with the score of 2.2750 out of 5. The score of this obstacle was considered as a low level of effects on the participants' reactions, attitudes and use of computer technologies. Hence, this gives emphasis to the positive attitudes of the participants toward the use of computer technologies. Additionally, the participants also had positive interest, PU, and PEOU to employ computer technologies in learning English. The obstacles that restricted the employment of computer technologies were linked to the external sources, such as the physical environment, and equipment.

## Conclusion

In ESL/EFL learning contexts, it is an evident that computer technologies have been viewed and realized as significant and useful learning tools especially among the ESL/EFL learners at schools and universities. With the quick globalization in the field of education, English language has become a vital factor for the academic and professional success of students whose first language is not English. In Saudi Arabia, where English is studied as a foreign language, Saudi EFL learners have realized the importance of using computer technologies in learning English. Mishal Al Shammari (2007) asserts that Saudi EFL students have tendency to use computer technologies in learning English and demonstrated positive stands and reactions towards the general use of CALL as an effective technique to develop the process of learning English. Consequently, the results of the current study are useful and beneficial for both students and teachers in schools and universities. With regard to students, they need to be exposed to computer tools to equip themselves such as motivation, skill, experience, self-confidence, and positive attitudes to participate in developing the process of learning English. As for teachers, they should offer assistance to students to encourage their perception of the computer tools. The teachers and facilitators should adopt a comprehensive method toward the teaching of computer tools to enable their students to meet their own strategic needs in English language learning. Thus, the findings of the present study enabled the researchers to draw up several useful implications in an attempt to improve the use of computer technologies among the Saudi EFL learners.
